# Role of Proton Motive Force in Photoinduction of Cytoplasmic Streaming in *Vallisneria* Mesophyll Cells

**DOI:** 10.3390/plants9030376

**Published:** 2020-03-18

**Authors:** Akiko Harada, Yoshiji Okazaki, Toshinori Kinoshita, Reiko Nagai, Shingo Takagi

**Affiliations:** 1 Department of Biology, Osaka Medical College, Takatsuki, Osaka 569-8686, Japan; bio004@osaka-med.ac.jp (Y.O.); 2 Division of Biological Science, Graduate School of Science, Nagoya University, Chikusa, Nagoya 464-8602, Japan; kinoshita@bio.nagoya-u.ac.jp; 3 Institute of Transformative Bio-Molecules (WPI-ITbM), Nagoya University, Chikusa, Nagoya 464-8602, Japan; 4 Department of Biological Sciences, Graduate School of Science, Osaka University, Toyonaka, Osaka 560-0043, Japan

**Keywords:** H^+^ motive force, plasma membrane H^+^-ATPase, photosynthesis, cytoplasmic streaming, Ca^2+^, *Vallisneria*

## Abstract

In mesophyll cells of the aquatic monocot *Vallisneria*, red light induces rotational cytoplasmic streaming, which is regulated by the cytoplasmic concentration of Ca^2+^. Our previous investigations revealed that red light induces Ca^2+^ efflux across the plasma membrane (PM), and that both the red light-induced cytoplasmic streaming and the Ca^2+^ efflux are sensitive to vanadate, an inhibitor of P-type ATPases. In this study, pharmacological experiments suggested the involvement of PM H^+^-ATPase, one of the P-type ATPases, in the photoinduction of cytoplasmic streaming. We hypothesized that red light would activate PM H^+^-ATPase to generate a large H^+^ motive force (PMF) in a photosynthesis-dependent manner. We demonstrated that indeed, photosynthesis increased the PMF and induced phosphorylation of the penultimate residue, threonine, of PM H^+^-ATPase, which is a major activation mechanism of H^+^-ATPase. The results suggested that a large PMF generated by PM H^+^-ATPase energizes the Ca^2+^ efflux across the PM. As expected, we detected a putative Ca^2+^/H^+^ exchange activity in PM vesicles isolated from *Vallisneria* leaves.

## 1. Introduction

Intracellular movements are closely associated with a wide spectrum of plant cell activities, including cell division [1,2,3], cell growth [4,5], redistribution of cell organelles [6,7], organized trafficking of membrane vesicles [3,8], and so on. Actin filaments and microtubules are representative cytoskeletal components that support a variety of unique motile machineries. The actin cytoskeleton is known to play pivotal roles in the regulation of intracellular movements, especially in response to environmental fluctuation, through its tremendously flexible nature [9,10]. However, the signaling pathways involved between the perception of environmental stimuli and the modulation of modes of intracellular movements are still not completely understood.

In muscle cells, Ca^2+^-sensitive regulatory mechanisms for the actomyosin-dependent generation of motive force have been extensively investigated, and a number of actin-linked and myosin-linked components responsible for the Ca^2+^ sensitivity have been identified [11,12]. Moreover, it is well documented that Ca^2+^ fluxes across the plasma membrane (PM) and the sarcoplasmic reticulum membrane of those motile cells function to control the cytoplasmic concentration of Ca^2+^ and excitation-contraction coupling [13]. The cytoplasmic streaming in internodal cells of Characean plants is actomyosin-dependent and regulated by Ca^2+^ [14]. In response to electrical or mechanical stimuli [15] or to hydration of the cytoplasm [15,16], a prompt cessation of cytoplasmic streaming is induced by a rapid increase in the cytoplasmic concentration of Ca^2+^. The sources of Ca^2+^ are different in each stimulus-dependent response: an influx of Ca^2+^ across the PM is induced by electrical or mechanical stimuli [17,18], while unidentified endomembranes serve as the Ca^2+^ source in the case of cytoplasm hydration [19]. Although knowledge has been accumulating about the molecular components of actin-based motile machineries in plant cells [20,21], dissection of regulatory mechanisms for intracellular movements have been largely hampered, especially in vascular plants.

We have been investigating the regulatory mechanism for light-sensitive rotational cytoplasmic streaming in mesophyll cells of the aquatic angiosperm *Vallisneria* [9,22]. Since the cytoplasmic streaming is induced by continuous illumination with red light and inhibited either by far-red light or by photosynthesis inhibitors [23,24], phytochrome and photosynthesis may cooperatively participate in the regulation. As demonstrated in Characean intermodal cells, the cytoplasmic streaming in *Vallisneria* mesophyll cells is also actomyosin-dependent [22,25,26,27], and regulated by Ca^2+^ [28]. By electron microscopic cytochemistry, we confirmed that the cytoplasmic content of calcium decreased in a mobile cytoplasm under red light and increased in an immobile cytoplasm under far-red light [23]. When protoplasts prepared from *Vallisneria* mesophyll cells were continuously illuminated with red light, the extracellular concentration of Ca^2+^ increased [29]. The effect of red light was antagonized either by far-red light or by the photosynthesis inhibitors [24]. Both the red light-induced efflux of Ca^2+^ and induction of cytoplasmic streaming were sensitive to vanadate, a general inhibitor for P-type ion-translocating ATPases, whereas blockers of PM Ca^2+^ channels substantially suppressed the far-red light-induced influx of Ca^2+^ and inhibition of cytoplasmic streaming [23,29]. Consequently, we hypothesized that Ca^2+^ transport systems across the PM are crucial for light-dependent changes in the cytoplasmic concentration of Ca^2+^, which in turn bring about the induction and cessation of cytoplasmic streaming.

In general, Ca^2+^-ATPase and Ca^2+^/H^+^ exchange activities play a central role in Ca^2+^ homeostasis in plant cells [30,31]. On the other hand, it has increasingly become evident that the plant master enzyme PM H^+^-ATPase is integrated in multiple signaling pathways derived from environmental cues [32]. Since we previously succeeded in detecting the activities of both the PM Ca^2+^-ATPase [22] and PM H^+^-ATPase [33,34], in this study we aimed to clarify which activity is predominantly involved in the photoinduction of Ca^2+^ efflux across the PM. Based on the results obtained from pharmacological, electrophysiological, and biochemical approaches, we propose that photosynthesis-dependent activation of PM H^+^-ATPase generates a large H^+^ motive force, which provides a driving force for the Ca^2+^ efflux across the PM.

## 2. Results

### 2.1. Effects of Metabolic Inhibitors on Photoinduction of Cytoplasmic Streaming

**Figure 1:**
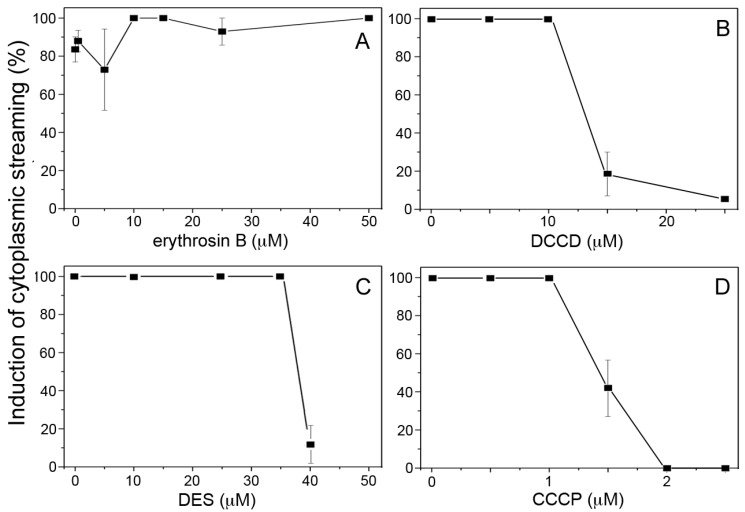
Effects of inhibitors on the photoinduction of cytoplasmic streaming in mesophyll cells of *Vallisneria*. Specimens in which no mesophyll cell exhibited cytoplasmic streaming after dark adaptation for 12 to 18 h were continuously illuminated with red light (λ_max_ = 650 nm, 10 µmol m^−2^ s^−1^). The ratios of the number of streaming cells to the total number of cells observed at 30 min after the start of illumination with red light were plotted as percentages against the concentration of erythrosin B (**A**), *N,N*′-dicyclohexylcarbodiimide (DCCD) (**B**), diethylstilbestrol (DES) (**C**) and carbonyl cyanide *m*-chlorophenyl hydrazone (CCCP) (**D**). The vertical bar of each point is standard error (SE) from the observation of about 100 cells out of a total of 3 to 22 different specimens. In the presence of each reagent at higher concentrations, namely, over 40 µM DCCD, 50 µM DES, and 4 µM CCCP, respectively, most cells appeared to be abnormal in which the chloroplasts were considerably deformed.

We first examined the effects of different kinds of inhibitors used for ion transport systems across the PM on the photoinduction of cytoplasmic streaming. In the control specimens, the cytoplasmic streaming was induced in all mesophyll cells after 20 to 30 min of continuous illumination with red light (650 nm, 10 µmol m^−2^ s^−1^), as described previously [23]. Erythrosin B is known as a specific inhibitor for plant PM Ca^2+^-ATPase when used at submicromolar concentrations [35,36,37]. Unexpectedly, red light induced nearly normal cytoplasmic streaming in the presence of erythrosin B at 0–50 µM (Figure 1A). We confirmed that erythrosin B at 0.5 µM substantially inhibited the ATP-dependent transport of Ca^2+^ but not that of H^+^ in the PM vesicles isolated from *Vallisneria* leaves (Figure S1).

In contrast, inhibitors for H^+^-ATPase, *N,N*′-dicyclohexylcarbodiimide (DCCD) and diethylstilbestrol (DES) [38,39,40], almost completely impaired the photoinduction of cytoplasmic streaming at 25 µM and 40 µM, respectively (Figure 1B,C). These results suggest that PM H^+^-ATPase rather than PM Ca^2+^-ATPase is responsible for induction of Ca^2+^-regulated cytoplasmic streaming. The H^+^ uncoupler, carbonyl cyanide *m*-chlorophenyl hydrazone (CCCP) at 2 µM also inhibited the photoinduction of cytoplasmic streaming (Figure 1D), further supporting the involvement of the H^+^ gradient (ΔpH) across the PM in the response.

### 2.2. Effects of Metabolic Inhibitors on Generation of H^+^ Motive Force

**Figure 2:**
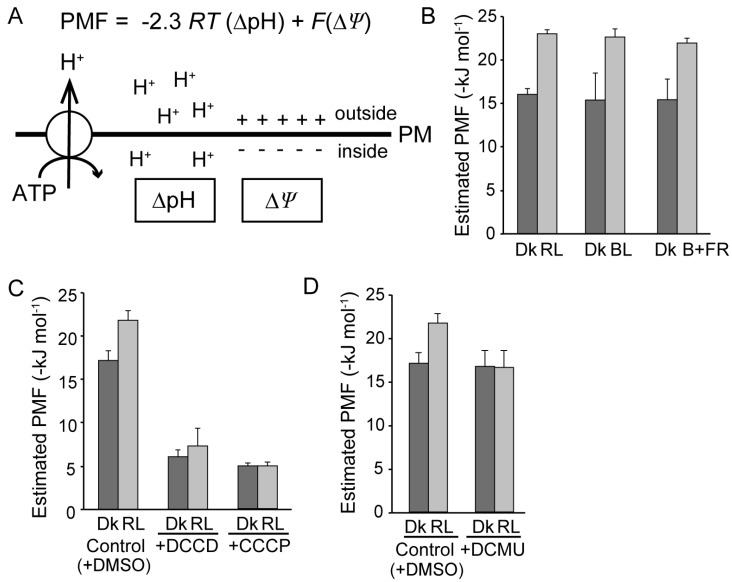
Estimated H^+^ motive force (PMF) before and after light illumination of *Vallisneria* mesophyll cells. (**A**) Scheme shows the PMF, which composed of a membrane potential (Δ*Ψ*) and a H^+^ activity gradient (ΔpH). Plasma membrane (PM) H^+^-ATPase activity contributes to generate the PMF (see the details in Materials and Methods). PM; plasma membrane. (**B**) Estimated PMF before (Dk) and after illumination with red (RL, 10 µmol m^−2^ s^−1^), blue (BL, 10 µmol m^−2^ s^−1^) and blue (10 µmol m^−2^ s^−1^) plus far-red light (10 µmol m^−2^ s^−1^) (B + FR). (**C**) Effects of 10 µM DCCD and 2.0 µM CCCP on the PMF before (Dk) and after illumination with red light (RL). (**D**) Effects of 10 µM 3-(3,4-dichlorophenyl)-1,1-dimethylurea (DCMU) on the PMF before (Dk) and after illumination with red light (RL). 0.5% dimethyl surfoxide (DMSO), a solvent for DCMU, was used as a control. PMF was estimated using the measured and published values of membrane potential and cytosolic pH from mesophyll cells of *Vallisneria* and epidermal cells of *Egeria densa*, listed in Table S1. Values are means ± SE. Each number of data is indicated in Table S1.

PM H^+^-ATPase generates the H^+^ motive force (PMF) across the PM, which is comprised of chemical and electrical components, namely, the H^+^ activity gradient (ΔpH) and the membrane potential (Δ*Ψ*, Figure 2A). Assuming that the PMF plays an important role in the photoinduction of cytoplasmic streaming, we estimated the magnitude of PMF under different conditions. For the estimation (Table S1), published values of cytosolic pH in *Egeria densa* epidermal cells [41] or *Phaeoceros laevis* gametophyte cells [42], either of those values gave the same estimation results, were used together with measured values of membrane potential in *Vallisneria* mesophyll cells [33]. While PMF was estimated to be about −16 kJ mol^−1^ in dark-adapted cells, it increased to −23 kJ mol^−1^ after 20 min of continuous illumination with red light (Figure 2B, Figure S2A). DCCD and CCCP decreased the PMF of dark-adapted cells to −6 kJ mol^−1^, and moreover, red light never increased the PMF in the presence of these inhibitors (Figure 2C).

On the other hand, a similar magnitude of PMF was generated by continuous illumination with blue light (446 nm, 10 µmol m^−2^ s^−1^), even when far-red light (729 nm, 10 µmol m^−2^ s^−1^) was superimposed in order to minimize the amount of Pfr, an active form of phytochrome (Figure 2B). An inhibitor of photosynthetic electron flow, 3-(3,4-dichlorophenyl)-1,1-dimethylurea (DCMU) at 10 µM did not affect PMF in dark-adapted cells, whereas it completely suppressed both the red- and blue-light-induced increase in PMF (Figure 2D, Figure S2B). Taken together, in *Vallisneria* mesophyll cells, we can assume that a large, photosynthesis-dependent PMF is generated through the enhanced activity of PM H^+^-ATPase.

### 2.3. Phosphorylation of the Penultimate Residue, Threonine, of the PM H^+^-ATPase in a Photosynthesis-dependent Manner

We previously demonstrated that the activities of ATP-dependent H^+^ transport and ATP hydrolysis in the PM fraction isolated from *Vallisneria* leaves are accelerated in a photosynthesis-dependent manner [33]. Phosphorylation of the penultimate residue, threonine (penultimate Thr), of the PM H^+^-ATPase and subsequent binding of a 14-3-3 protein activates the PM H^+^-ATPase in response to a wide variety of biotic and abiotic stimuli [32,43]. In this study, we asked whether the same activation mechanism is involved in photosynthesis-dependent activation of PM H^+^-ATPase in *Vallisneria* mesophyll cells.

**Figure 3:**
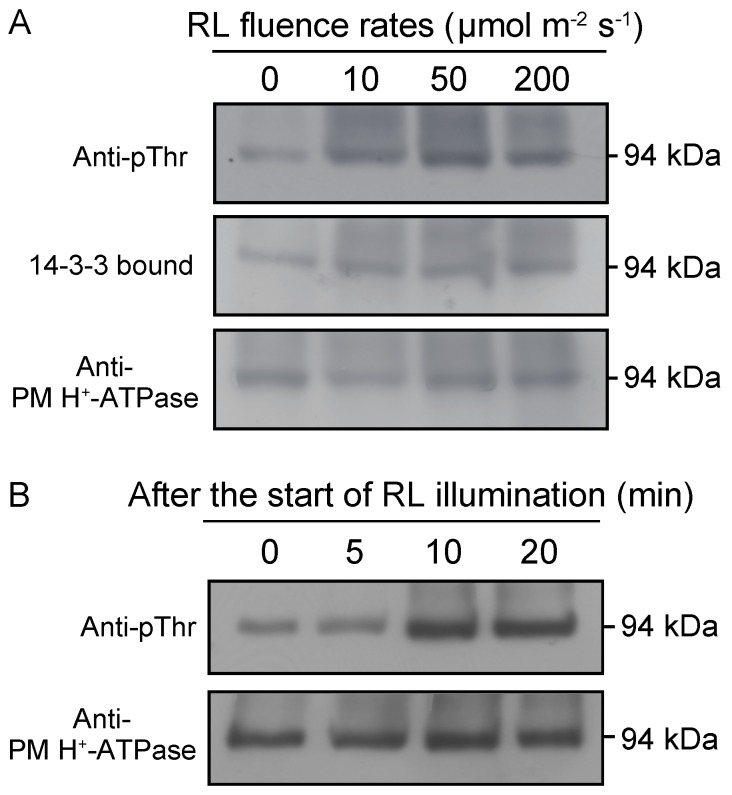
Phosphorylation of the PM H^+^-ATPase in *Vallisneria* leaves in response to red light. (**A**) Fluence rate-dependent phosphorylation of PM H^+^-ATPase and the binding of 14-3-3 protein. Dark-adapted leaves were illuminated with 0, 10, 50 and 200 µmol m^−2^ s^−1^ of red light for 10 min. Thephosphorylation status and amount of PM H^+^-ATPase were determined by immunoblot analysis using anti-pThr antibodies (top; Anti-pThr) and anti-PM H^+^-ATPase for *Arabidopsis* AHA2 (bottom, Anti-PM H^+^-ATPase), respectively. Binding of 14-3-3 protein was determined by protein-blot analysis using glutathione *S*-transferase (GST)-14-3-3 protein (*Arabidopsis* GF14phi) as a probe (middle; 14-3-3 bound). Data are representative of 2 independent experiments. (**B**) Time course of phosphorylation of the PM H^+^-ATPase in response to red light. Dark-adapted leaves were illuminated with red light at 10 µmol m^−2^ s^−1^ for 0, 5, 10, and 20 min after the start of illumination. The rest of the procedure was as described for (**A**). Data are representative of 2 independent experiments.

After dark-adapted leaves were illuminated with red light at different fluence rates, leaves were homogenized and the crude extracts were subjected to immunoblot analysis using two different kinds of antibodies: one raised against the conserved catalytic domain of *Arabidopsis* PM H^+^-ATPase AHA2 (anti-PM H^+^-ATPase; [44]) and the other against the phosphorylated penultimate Thr-947 of AHA2 (anti-pThr; [44]). Both antibodies detected a 94-kDa band (Figure 3A), which is likely identical to the polypeptides cross-reacted with antibodies raised against the central consensus loop of tobacco PM H^+^-ATPase PMA1 [34]. Immunoblotting with anti-pThr clearly showed that red light at higher fluence rates than 10 µmol m^−2^ s^−1^ induced phosphorylation of the 94-kDa polypeptides (Figure 3A). Furthermore, protein blot analysis using recombinant 14-3-3 protein (*Arabidopsis* 14phi) as a probe revealed that the phosphorylated 94-kDa polypeptides bound to the 14-3-3 protein (Figure 3A). The amount of 94-kDa polypeptides appeared to be constant irrespective of red-light illumination (Figure 3A).

Phosphorylation of the 94-kDa polypeptides became obvious at 10 min of continuous illumination with red light at 10 µmol m^−2^ s^−1^ (Figure 3B). This time course matches that of light-induced membrane hyperpolarization, which reached its maximum rate at 10–20 min of illumination (Figure S3, [33]), and that of photoinduction of cytoplasmic streaming [23].

**Figure 4:**
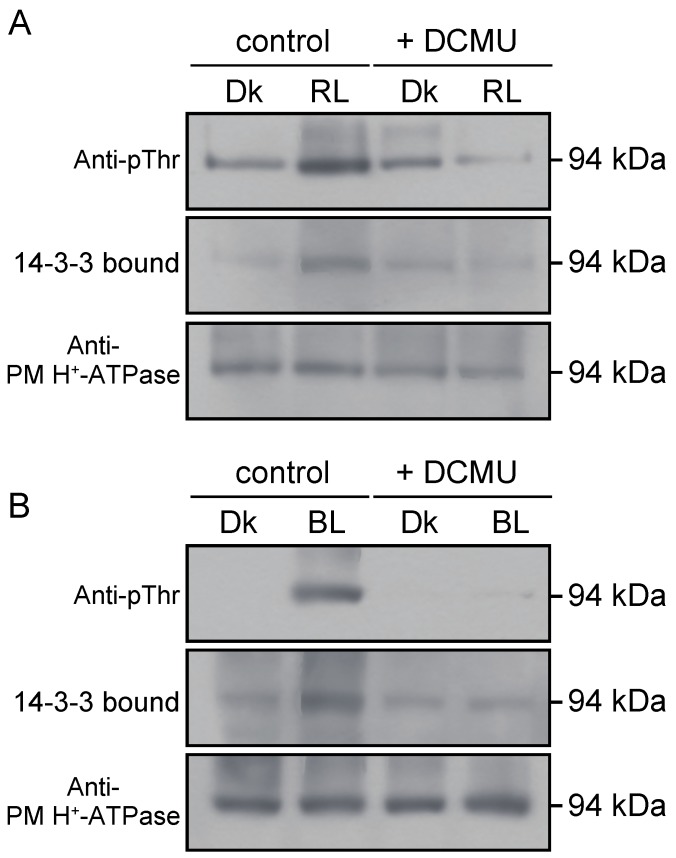
Involvement of photosynthesis in the light-induced phosphorylation of PM H^+^-ATPase in *Vallisneria* leaves. Dark-adapted leaves were pre-treated with 10 µM DCMU (+ DCMU) or with 0.1% DMSO, a solvent of DCMU (control), for 40 min and then illuminated with 10 µmol m^−2^ s^−1^ of red light (**A**, RL) or blue light (**B**, BL) for 10 min or kept in the dark (Dk). The rest of the procedure was as described for Figure 3.

Both red and blue light induced phosphorylation of the 94-kDa polypeptides and the binding to 14-3-3 protein (Figure 4). These responses were completely inhibited by DCMU at 10 µM (Figure 4), indicating the crucial involvement of photosynthesis in the regulation. 

### 2.4. Ca^2+^/H^+^ Exchange Activity in the PM Vesicles

**Figure 5:**
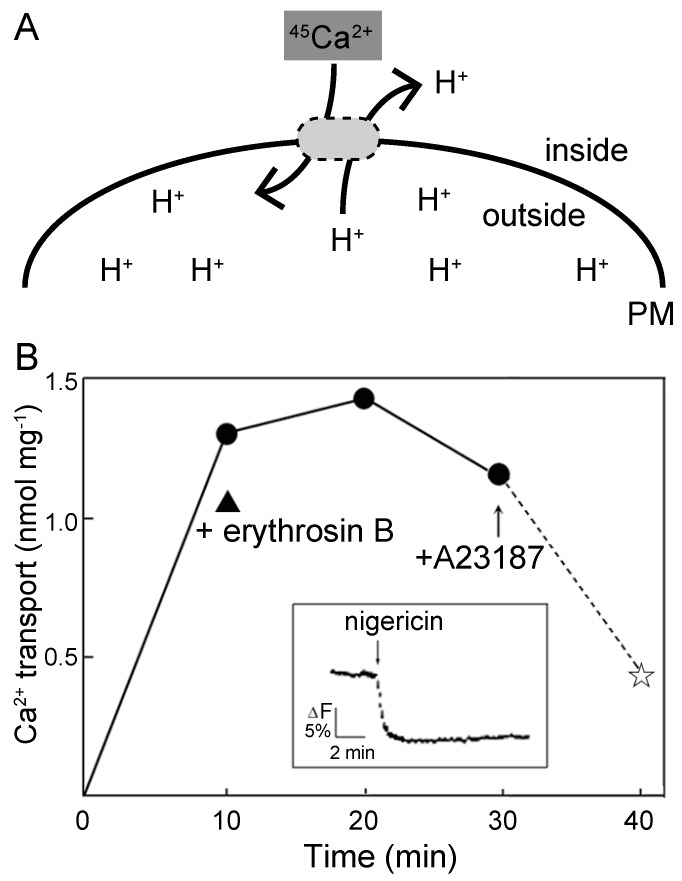
ATP-independent exchange of Ca^2+^ with H^+^ preloaded into the inside-out PM vesicles prepared from *Vallisneria* leaves. (**A**) Schematic diagram of the experiment. Transport of exogenously added radioactive Ca^2+^, ^45^Ca^2+^, into the H^+^-loaded inside-out PM vesicles was examined. Transported ^45^Ca^2+^ was measured by a membrane filtration method. PM; plasma membrane of inside-out vesicles. (**B**) After inside-out vesicles had been prepared in the presence of 125 mM of KCl, nigericin was added to induce an influx of H^+^ into the vesicles (Inset, the ordinate is the relative intensity of fluorescence from quinacrine). Transport of exogenously added Ca^2+^ into the H^+^-loaded vesicles was examined in the absence of ATP. The activity in the presence of 0.5 µM erythrosine B is also indicated (closed triangle). A23187 at a final concentration of 5 µM was added (arrow) and the activity after addition of A23187 is indicated as a star.

An efflux of Ca^2+^ across the PM is closely related to the photoinduction of cytoplasmic streaming [24,27]. Assuming that the large PMF provided the driving force for Ca^2+^ efflux across the PM to induce Ca^2+^-regulated cytoplasmic streaming, we lastly attempted to detect Ca^2+^/H^+^ exchange activity using isolated PM vesicles with inside-out sidedness, which are postulated to have an increased membrane tightness to H^+^ [45]. We first attempted to detect an influx of exogenously added Ca^2+^ into the H^+^-loaded inside-out PM vesicles (Figure 5A). Using the method of Kasai and Muto [46], K^+^-loaded inside-out PM vesicles were treated with nigericin to induce an exchange of loaded K^+^ with H^+^ (inset in Figure 5B). The subsequent addition of radioactive Ca^2+^ induced a significant amount of Ca^2+^ influx into the inside-out vesicles in the absence of ATP (Figure 5B). The detected activity at 10 min was 0.13 nmol Ca^2+^ mg^−1^ min^−1^, which corresponds to 20% of the total activity of ATP-dependent Ca^2+^ transport of 0.65 nmol Ca^2+^ mg^−1^ min^−1^ [22]. The transported Ca^2+^ was released upon the addition of a divalent cationophore A23187 (Figure 5B, +A23187). Even in the presence of erythrosin B at 0.5 µM, a similar amount of Ca^2+^ influx was detectable (Figure 5B, + erythrosin B). In a duplicate experiment, the amount of ATP-independent Ca^2+^ transport at 10 min was 1.30 nmol Ca^2+^ mg^−1^, and it decreased to 0.45 nmol Ca^2+^ mg^−1^ after the addition of A23187. These results suggest that Ca^2+^ can enter PM vesicles by utilizing a ΔpH across the PM.

**Figure 6:**
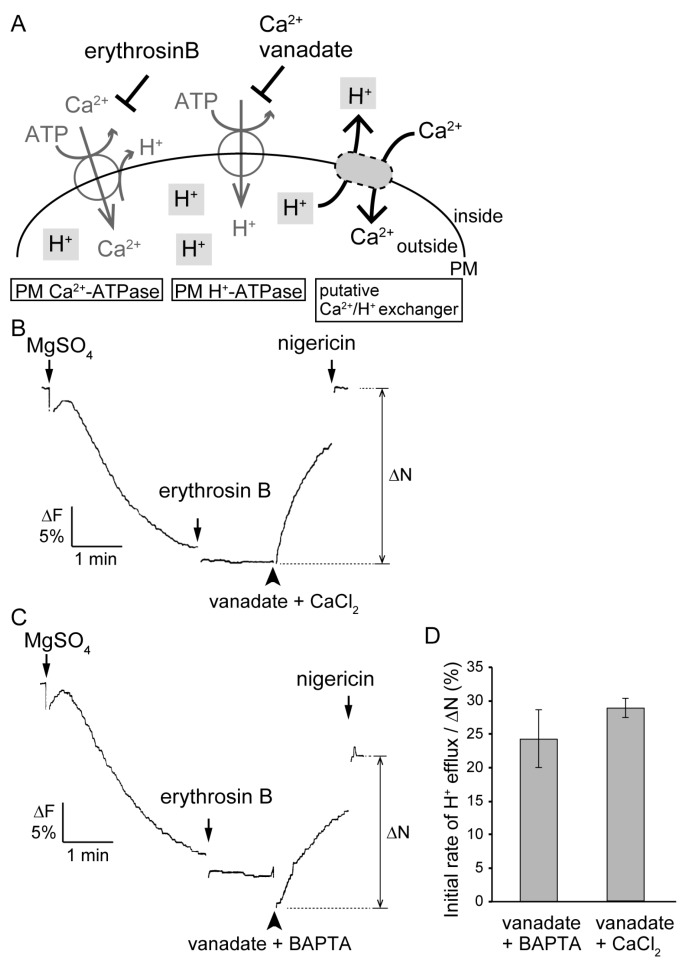
Ca^2+^-induced H^+^ efflux from the H^+^-loaded inside-out PM vesicles prepared from *Vallineria* leaves. (**A**) Schematic diagram of H^+^ efflux from H^+^-preloaded inside-out vesicles by exogenously added Ca^2+^. Left (PM Ca^2+^-ATPase); The addition of Ca^2+^ induces an efflux of H^+^ coupled with PM Ca^2+^-ATPase-driven influx of Ca^2+^ into the vesicles. Middle (PM H^+^-ATPase); The addition of Ca^2+^ induces passive leakage of H^+^ because of Ca^2+^ inhibition of PM H^+^-ATPase activity [47]. Right (putative Ca^2+^/H^+^ exchanger); The addition of Ca^2+^ induces H^+^ efflux through an unidentified Ca^2+^/H^+^ exchanger. The H^+^ efflux coupled with PM Ca^2+^-ATPase activity (left) and the passive H^+^ leakage caused by inhibition of PM H^+^-ATPase activity (middle) can be eliminated by erythrosin B and vanadate, respectively. PM; plasma membrane of inside-out vesicles. (**B**) H^+^ efflux induced by exogenously added vanadate with Ca^2+^. ATP-dependent H^+^ uptake into inside-out PM vesicles in a reaction medium containing 2.0 mM ATP was started by addition of 5 mM MgSO_4_. After the reaction was saturated, 0.5 µM erythrosine B was added, and then 200 µM vanadate was further added with 30 µM CaCl_2_ (vanadate + CaCl_2_). Finally the H^+^ gradient across the PM was collapsed by nigericin, a H^+^/K^+^ exchanger, to confirm that the membrane vesicles were sealed. (**C**) H^+^ efflux induced by exogenously added vanadate without Ca^2+^. Following the addition of erythrosine B, 200 µM vanadate was added with 2 mM O,O’-bis(2-aminophenyl)ethyleneglycol-N,N,N′,N′-tetraacetic acid (BAPTA) (vanadate + BAPTA). The rest of the experiments were conducted as described for (B). The ordinate is the relative intensity of fluorescence from quinacrine. (**D**) The average of initial rate of H^+^ efflux by exogenously added vanadate with BAPTA and with CaCl_2_, standardized by recovery of quinacrine fluorescence by addition of nigericin (∆N) (n = 2). Vertical bars indicate SE.

We had already succeeded in detecting active H^+^ transport into the inside-out PM vesicles isolated from the *Vallisneria* leaves by measuring the quenching rate of fluorescence from quinacrine [33]. Using this experimental system, we next aimed to detect H^+^ efflux from H^+^-loaded inside-out PM vesicles upon exogenous application of Ca^2+^. H^+^ efflux which is dependent on the putative Ca^2+^/H^+^ exchange activity (Figure 6A, right) should be distinguished from that associated with the operation of PM Ca^2+^-ATPase [35] (Figure 6A, left) and the passive leak of H^+^ due to the direct inhibition of PM H^+^–ATPase by the addition of Ca^2+^ [47] (Figure 6A, middle).

We confirmed the occurrence of H^+^ efflux associated with the operation of PM Ca^2+^-ATPase using the method of Rasi-Caldogno [36]. After a saturating ATP-dependent transport of H^+^ into the inside-out PM vesicles, the addition of CaCl_2_ to a final concentration of 30 µM induced a rapid collapse of the H^+^ gradient (Figure S4). The subsequent addition of erythrosin B significantly retarded the rate of decay of the H^+^ gradient. These results suggest that PM Ca^2+^-ATPase of *Vallisneria* leaves shares the characteristics of an enzyme that catalyzes nH^+^/Ca^2+^ exchange as demonstrated in radish seedlings [35] and *Sinapis* root hairs [48], and that H^+^ efflux associated with the operation of PM Ca^2+^-ATPase could be maximally suppressed in the presence of erythrosin B.

In the next experiment, we used erythrosin B and vanadate to inhibit the activities of PM Ca^2+^–ATPase and PM H^+^–ATPase, respectively. Erythrosin B at 0.5 µM inhibited the activity of ATP-dependent Ca^2+^ transport to 14% (Figure S1). Vanadate at 200 µM, with CaCl_2_ at 30 µM or with a Ca^2+^-chelating reagent O,O’-bis(2-aminophenyl)ethyleneglycol-N,N,N′,N′-tetraacetic acid (BAPTA) at 2 mM, inhibited ATP-dependent H^+^ transport to the same level of about 9% (Figure S5). After a saturating ATP-dependent transport of H^+^ into the inside-out PM vesicles, the addition of erythrosin B did not affect the fluorescence level (Figure 6B,C). We then added vanadate with CaCl_2_ (Figure 6B) or with BAPTA (Figure 6C). Both treatments induced instantaneous efflux of H^+^ from the PM vesicles; a faster decay of the H^+^ gradient was consistently observed in the presence of Ca^2+^, though BAPTA seemed to decrease both the basal level of the quinacrine fluorescence and its recovery after the addition of nigericin (∆N) (Figure 6C). The initial rate of change in the level of quinacrine fluorescence per 15 sec standardized by the magnitude of ∆N (Figure 6B, C) were 28.8 ± 1.4% and 24.3 ± 4.3% in the presence and absence of Ca^2+^, respectively (n = 2, Figure 6D). When vanadate at 200 µM alone was added, the rate was 25.2 ± 0.6% (n = 2). Since H^+^ efflux associated with the operation of PM Ca^2+^–ATPase should be negligible in the presence of erythrosin B (Figure 6A, left), the Ca^2+^-dependent difference in the rate of decay of H^+^ gradient could be ascribed to Ca^2+^/H^+^ exchange (Figure 6A, right). Taken together, these results support our idea that Ca^2+^ and H^+^ are exchanged using the H^+^ gradient across the PM in *Vallisneria* mesophyll cells.

## 3. Discussion

### 3.1. Large PMF is Necessary for the Photoinduction of Cytoplasmic Streaming

Based on the present results, we propose that PM H^+^-ATPase plays crucial roles in the photoinduction of cytoplasmic streaming in mesophyll cells of *Vallisneria*. Since the cytoplasmic streaming is regulated by the cytoplasmic concentration of Ca^2+^ [28], and both the red light-induced cytoplasmic streaming and the Ca^2+^ efflux were sensitive to vanadate [29], we first postulated an involvement of PM Ca^2+^-ATPase in both responses. The photoinduction of cytoplasmic streaming, however, is insensitive to erythrosin B (Figure 1A), which predominantly inhibits the ATP-dependent transport of Ca^2+^ but not that of H^+^ in the PM vesicles isolated from *Vallisneria* leaves (Figure S1). Although we could not determine whether erythrosin B permeates into the mesophyll cells in the present study, Felle et al. [48] demonstrated by electrophysiological procedures that exogenously applied erythrosin B inhibited the PM Ca^2+^-ATPase in living root hair cells.

On the other hand, the inhibitors for H^+^-ATPase (DCCD and DES) and the uncoupler (CCCP), which drastically decreased PMF (Figure 2C), suppressed the photoinduction of cytoplasmic streaming in a concentration-dependent manner (Figure 1B–D). It is widely accepted that a large PMF energizes multiple ion channels and secondary transporters. Believing that a large PMF under red light provides the driving force for Ca^2+^ efflux, we succeeded in detecting the exchange activity of H^+^ with Ca^2+^ across the PM using PM vesicles isolated from the leaves (Figure 5 and Figure 6). Taken together, the increased PMF may drive Ca^2+^ efflux during photoinduction of Ca^2+^-regulated cytoplasmic streaming. The molecular basis for Ca^2+^/H^+^ exchange activity across the PM should be investigated in the near future.

### 3.2. Photosynthesis-dependent Phosphorylation of the Penultimate Residue, Thr, of PM H^+^-ATPase is Involved in the Generation of PMF

In this study, we found that photosynthesis controls the phosphorylation status of the penultimate Thr of PM H^+^-ATPase (Figure 3 and Figure 4), which is known as a major activation mechanism of PM H^+^-ATPase [32,43]. Phosphorylation of the PM H^+^-ATPase and the generation of a large PMF were induced under red and blue light, exhibited almost identical time courses, and were sensitive to DCMU (Figure 2, Figure 3 and Figure 4, Figure S2). These results strongly suggest that the large PMF is generated by the activation of PM H^+^-ATPase by photosynthesis-dependent phosphorylation of the penultimate Thr.

Since photosynthetic control of phosphorylation of PM H^+^-ATPase is demonstrated in the thalli of the liverwort *Marchantia polymorpha* [49] and mesophyll cells of *A. thaliana* [50], we suppose that the mechanism is ubiquitously shared in photosynthesizing plants. We would like to propose that one of the possible roles of this mechanism is to induce cytoplasmic streaming in mesophyll cells, enabling efficient delivery of photosynthetic metabolites [51,52]. Thus a well-established activation mechanism, namely, phosphorylation of the penultimate Thr in PM H^+^-ATPase, is involved in signaling pathways induced by a wide spectrum of exogenous and endogenous stimuli such as blue light, red light, the phytohormones auxin, gibberellin, abscisic acid, and brassinosteroid, and sucrose in various types of cells [49,50,53,54,55,56,57,58]. Dissection of conserved as well as specific factors functioning in individual signaling pathways will deepen our understanding of the significance of this activation mechanism of PM H^+^-ATPase. 

### 3.3. Large PMF is Not Sufficient for the Photoinduction of Cytoplasmic Streaming

From the estimated magnitude of the PMF (Figure 2, Figure S2), we noticed that cytoplasmic streaming could be induced only when the PMF was larger than 20 kJ mol^−1^. However, that magnitude of PMF alone is not sufficient to induce cytoplasmic streaming. For example, blue-light illumination with or without superimposed far-red light increased the PMF to over 20 kJ mol^−1^ (Figure 2), but blue light alone is not able to induce cytoplasmic streaming [23]. We previously clarified that cytoplasmic streaming is induced only in the presence of Pfr and only when intact photosynthesis takes place [24]. Therefore, the absence of cytoplasmic streaming under blue light may be attributable to an insufficient amount of Pfr.

Regarding the role of Pfr, we have demonstrated that phytochrome is involved in the regulation of cytoplasmic motility in *Vallisneria* epidermal cells [59]. The cytoplasm is rendered quiescent in darkness, whereas its motility is rapidly activated upon light exposure in a red/far-red light reversible manner. In general, preceding the initiation of intracellular movements, the cytoplasmic matrix has to gain appropriate motility [60]. Although we suggested an involvement of Ca^2+^ in the photoregulation of cytoplasmic motility [59], a precise interrelationship between the regulation of cytoplasmic motility and the role of PMF in the photoinduction of cytoplasmic streaming remains to be elucidated.

## 4. Materials and Methods 

### 4.1. Plants

*Vallisneria* sp. was cultured as described in Izutani et al. [61] under a 12-h light and 12-h dark regimen.

### 4.2. Observation of Cytoplasmic Streaming

Specimens for light microscopy were prepared as described by Izutani et al. [61]. Briefly, pieces of a leaf were cut open in the middle of the layers of mesophyll cells. After floating in artificial pond water (APW), which contained 0.5 mM KCl, 0.2 mM NaCl, 0.1 mM Ca(NO_3_)_2_, 0.1 mM Mg(NO_3_)_2_, and 2 mM PIPES-NaOH at pH 7.0, for one cycle of the dark and light regimen, each half piece of the leaf was mounted on a glass slide with the mesophyll cell side downward. The glass slide was immersed in fresh APW and kept in complete darkness for another 12 to 18 h. After dark treatment, we confirmed that all the mesophyll cells did not show any sign of streaming (Supplementary movie). Then those cells were continuously illuminated with red light (650 nm, 10 µmol m^−2^ s^−1^) on the stage of a light microscope from below through a condenser lens, as previously described [23]. The photoinduction of cytoplasmic streaming was evaluated at 30 min of red light illumination as the ratio of the number of streaming cells, in which cytoplasmic particles exhibited continuous movement for at least 5 sec (Supplementary movie), to the total number of cells observed. When specimens were treated with metabolic inhibitors, half pieces of the leaf were floated in APW that contained each reagent for 1 h before being mounted on a glass slide and then kept in darkness for 12 to 18 h in APW supplemented with each reagent. DCCD, DES, and CCCP were dissolved in dimethyl surfoxide (DMSO) and then diluted 200-fold with APW. APW containing 0.5% (v/v) DMSO was used as a control. The effects of DCCD and DES may not be attributable to a possible decrease in the intracellular level of ATP [62], because we confirmed that cytoplasmic streaming can be induced by treatment with ethylene glycol-bis(2-aminoethylether)-*N,N,N′,N*′-tetraacetic acid (EGTA) at 10 mM in cells that had been illuminated with red light in the presence of DCCD at 25 µM or DES at 40 µM. 

### 4.3. Estimation of PMF

The PMF (Δ*µ*_H+_) was estimated by following equation:

Δ***µ*_H+_ = *µ*_H+_^in^_−_*µ*_H+_^out^**

**= *RT* ln (A_H+_^in^ / A_H+_^out^) + *z*_H+_*F* (**Δ***Ψ*)**

**= −2.3 *RT* (pH_cyt_ − pH_out_) + *F* (**Δ***Ψ*)**

**= −2.3 *RT* (ΔpH) + *F* (**Δ***Ψ*)**

where *R* is gas constant, *T* is absolute temperature, A_H+_^in^ is the cytosolic concentration of H^+^, A_H+_^out^ is the extracellular concentration of H^+^, *z* is valency of H^+^, *F* is Faraday’s constant, pH_cyt_ is cytosolic pH, pH_out_ is extracellular pH and Δ*Ψ* is the membrane potential. Values of membrane potential [33] and cytosolic pH [41,42] before and after light illumination are listed in Table S1. Extracellular pH was buffered to 7.0. In the presence of CCCP, cytosolic pH was assumed to be equal to extracellular pH. Membrane potential of mesophyll cells was measured according to Harada et al. [33].

### 4.4. Isolation of PM Fraction

A PM-rich fraction was prepared from the leaves according to Harada et al. [33] under white light. Briefly, healthy leaf segments were homogenized with a Polytron homogenizer (PT35/2ST”OD”; Kinematica, Luzern, Switzerland) in a homogenizing medium that contained 300 mM sucrose, 10 mM EGTA, 5 mM ethylenediamine-*N,N,N′,N*′-tetraacetic acid (EDTA), 5 mM K_2_S_2_O_5_, 1 mM dithiothreitol (DTT), 10 mg ml^−1^ butylated hydroxytoluene, 1% (w/v) casein, 1.2 mg ml^−1^ aprotinin, 2.5 mg ml^−1^ pepstatin, 20 mg ml^−1^ polyvinylpolypyrrolidone, and 50 mM MOPS-KOH at pH 7.6. After differential centrifugation, the resultant pellet was washed with buffer A (250 mM sucrose, 0.1 mM DTT, and 10 mM MOPS-KOH at pH 7.6) and designated as a crude microsome fraction. A PM fraction was isolated from the crude microsome fraction by an aqueous two-phase partitioning at pH 7.8 using a polymer mixture composed of dextran T500 (Amersham Pharmacia Biotech AB, Uppsala, Sweden) and polyethylene glycol P-3640 (Sigma, St. Louis, MO, USA). All the procedures were carried out at 0 to 4 °C.

### 4.5. Assay of the Ca^2+^/H^+^ Exchange Activity using Radioactive Ca^2+^

H^+^-loaded PM vesicles with inside-out orientation were prepared through a nigericin-mediated exchange of preloaded K^+^ with H^+^ as described by Kasai and Muto [46]. Briefly, the PM fraction was made in a buffered solution that contained 125 mM KCl instead of 250 mM sucrose, and was then incubated with 0.2% (w/v) Triton X-100 in the presence of 125 mM KCl for 10 min on ice. Since over 90% of the total activity of ATP hydrolysis was recovered after removal of Triton X-100, substantial impairment of ATPases in the PM by Triton X-100 did not occur under the present conditions. These K^+^-loaded inside-out vesicles were further treated with nigericin to induce an exchange of loaded K^+^ with H^+^. ^45^Ca^2+^ transport into the H^+^-loaded inside-out vesicles was measured by a membrane filtration method according to Bush et al. [63] in the absence of ATP.

### 4.6. Assay of the Ca^2+^-Induced H^+^ Efflux from H^+^-Loaded PM Vesicles

The activity of ATP-dependent H^+^ transport into inside-out PM vesicles was assayed at 30 °C according to Harada et al. [33]. Briefly, the inside-out PM vesicles were prepared by mixing a small aliquot of the suspended PM fraction in buffer B (250 mM sucrose, 1 mM DTT, 5 mM EDTA-bis-tris propane (BTP), and 10 mM MOPS-BTP at pH 7.0) at 1 mg ml^−1^ of protein with a 1% volume of each of 200 mM ATP-BTP at pH 7.0 and 1175 mg ml^−1^ Brij 58 [64]. The prepared vesicles were added to the reaction medium [65] that contained 10 µM quinacrine instead of acridine orange and was supplemented with 500 µg ml^−1^ Brij 58. After incubation for 5 min, the reaction was started by the addition of MgSO_4_ to a final concentration of 5 mM. The rate of quenching of fluorescence from quinacrine was monitored with a fluorescence spectrophotometer (Model 850; Hitachi, Tokyo, Japan). The excitation and emission wavelengths were 420 and 495 nm, respectively. Nigericin dissolved in ethanol was used at 1 µg ml^−1^ to dissipate the generated H^+^ gradient across the membrane of vesicles. Stock solutions of BAPTA, vanadate, and CaCl_2_ were buffered to pH 7.0 with 50 mM, 2 mM and 2 mM MOPS-BTP (pH 7.0), respectively.

### 4.7. Immunoblot and Protein-Blot Analysis

Immunoblot and protein blot analyses were performed according to previous methods [44,49,66] with minor modifications. Leaf segments kept in darkness for 12–16 h, were illuminated with or without red light (650 nm, 10 µmol m^−2^ s^−1^) or blue light (470 nm, 10 µmol m^−2^ s^−1^) were cut into small pieces with a razor blade and then homogenized in an ice-cold homogenization buffer (100 mM MOPS-KOH, pH7.5, 200 mM NaCl, 5 mM EDTA, 10 mM NaF, 1 mM DTT, and 20 µL/L protein inhibitor cocktail for plant cell lysate [Sigma-Aldrich, St. Louis, MO, USA]) using a mortar and pestle. The homogenate was solubilized by the addition of half the volume of sodium dodecyl sulfate (SDS) sample buffer (4.5% [w/v] SDS, 45% [w/v] sucrose, 15% [v/v] 2-mercaptoethanol, 0.012% [w/v] Coomassie Brilliant Blue, 1.5 mM EDTA, and 45 mM Tris-HCl [pH 8.0]). The solubilized samples were incubated at 52 °C for 10–15 min. Then the samples were centrifuged at 12,000g for 1 min and the resulting supernatant was subjected to SDS-PAGE. Red and blue light were obtained by a light-emitting diode (LED) red light illuminator (ISC-150x150-H4RR, CCS Inc., Kyoto, Japan) and a LED blue light illuminator (ISC-150x150-BB, CCS Inc., Kyoto, Japan), respectively.

The polyclonal antibodies raised against the catalytic domain of *Arabidopsis* AHA2 (anti-PM H^+^-ATPase), phosphorylated penultimate Thr-947 of AHA2 (anti-pThr), glutathione *S*-transferase (GST), and *Arabidopsis* GF14phi (anti-14-3-3 protein) were described previously [43,44]. Anti-PM H^+^-ATPase and anti-pThr recognize not only AHA2 but also other H^+^-ATPase isoforms in *Arabidopsis* [44]. For protein blots, we used GF14phi protein fused to GST as a probe.

## Supplementary Materials

The following are available online at https://www.mdpi.com/2223-7747/9/3/376/s1, Table S1: Membrane potential (Δ*Ψ*) and cytosolic pH (pH_cyt_) under dark and light for estimation of PMF, Figure S1: Effects of erythrosin B on the ATP-dependent Ca^2+^ and H^+^ transport activity in the PM vesicles isolated from *Vallisneria* leaves, Figure S2: Estimated PMF before and after light illumination of *Vallisneria* mesophyll cells, Figure S3: Red light-induced hyperpolalization of membrane potential in a *Vallisneria* mesophyll cell, Figure S4: Ca^2+^-induced H^+^ efflux from the H^+^-loaded inside-out PM vesicles prepared from *Vallisneria* leaves, Figure S5: Inhibitory effects of vanadate with BAPTA or with Ca^2+^ on the ATP-dependent H^+^ transport activity in inside-out PM vesicles prepared from *Vallisneria* leaves, Supplementary movie: Induction of cytoplasmic streaming in response to white light in *Vallisneria* mesophyll cells.
