# Supplementary material for: Role of Proton Motive Force in Photoinduction of Cytoplasmic Streaming in Vallisneria Mesophyll Cells
Source: Plants (Basel). 2020 Mar 18;9(3):376. doi: 10.3390/plants9030376 (PMC7154820; doi:10.3390/plants9030376)
Supplement: Supplementary file 1 [file plants-09-00376-s001.zip › supplemental materials (after proofreading)/Legend for Supplementary Movie.docx]

Supplementary Movie

Induction of cytoplasmic streaming in response to white light in *Vallisneria* mesophyll cells.

The movie is reproduced at 20x speed. After dark treatment for 12-18 hr, no mesophyll cells exhibited cytoplasmic streaming under safe light (green light). Continuous illumination with white light (200 μmol m^-2^ s^-1^) induced cytoplasmic streaming. Mesophyll cells are indicated by dashed red lines at the beginning of the video. The smaller cells in the background are epidermal cells. Green particles in the cells are chloroplasts. The start of the illumination is indicated as “White light ON”.
