# Supplementary material for: Role of Proton Motive Force in Photoinduction of Cytoplasmic Streaming in Vallisneria Mesophyll Cells
Source: Plants (Basel). 2020 Mar 18;9(3):376. doi: 10.3390/plants9030376 (PMC7154820; doi:10.3390/plants9030376)
Supplement: Supplementary file 1 [file plants-09-00376-s001.zip › supplemental materials (after proofreading)/plants-721360-supplementary-2.doc]

TableS1. Membrane potential (Δ*Ψ*) and cytosolic pH (pH_cyt_) under dark and light for estimation of PMF.

| light | treatment | n | Δ*Ψ*(dark) (mV) | pH_cyt_ (dark) | Δ*Ψ*(light) (mV) | pH_cyt_ (light) |
| --- | --- | --- | --- | --- | --- | --- |
| RL | - | 4 | -155.0 ± 6.7 | 7.2 ^a)^ | -232.0 ± 4.7 | 7.1 ^a)^ |
| BL | - | 3 | -147.7 ± 32.4 | 7.2 ^a)^  6.8 ^b)^ | -228.7 ± 10.4 | 7.1 ^a)^  6.7 ^b)^ |
| RL | Control (DMSO) | 5 | -166.6 ± 12.3 | 7.2 ^a)^ | -220.0 ± 11.7 | 7.1 ^a)^ |
|  | + DCCD | 3 | -68.6 ± 8.3 | 6.9 ^a)^ | -81.3 ± 21.0 | 6.9 ^a)^ |
|  | + CCCP | 5 | -51.0 ± 19.0 | 7.0 ^c)^ | -51.2 ± 4.2 | 7.0 ^c)^ |
| RL | Control (DMSO) | 5 | -166.6 ± 12.3 | 7.2 ^a)^ | -220.0 ± 11.7 | 7.1 ^a)^ |
|  | + DCMU | 7 | -162.3 ± 19.4 | 7.2 ^a)^ | -161.9 ± 19.2 | 7.2 ^a)^ |
| BL | Control (DMSO) | 7 | -153.6 ± 9.2 | 7.2 ^a)^ | -226.6 ± 8.5 | 7.1 ^a)^ |
|  | + DCMU | 1 | -129 | 7.2 ^a)^ | -137 | 7.2 ^a)^ |

Δ*Ψ*(dark): dark-adapted level of membrane potential. pH_cyt_ (dark): cytosolic pH under dark. Δ*Ψ*(light): maximum hyperpolarized level of membrane potential after light illumination. pH_cyt_ (light): cytosolic pH after light illumination. Values of membrane potentials are means ± SE.

^a)^ Published values of cytosolic pH of epidermal cells of *Egeria densa* [41]. ^b)^ Published values of cytoplasmic pH of gametophyte cells of *Phaeoceros laevis* [42]. ^c)^ In the presence of CCCP, cytosolic pH was assumed to be equal to extracellular pH, 7.0. + DCCD: pretreatment with 25 µM DCCD for 3 h before light illumination. + DCMU: pretreatment with 10 µM DCMU for 30 min before light illumination. Control: 0.5% (v/v) DMSO, a solvent for DCCD and DCMU was used as control. Extracellular pH was buffered to 7.0. n: number of cells measured.

Figure S1. Effects of erythrosin B on the ATP-dependent Ca^2+^ and H^+^ transport activity in the PM vesicles isolated from *Vallisneria* leaves. We measured ATP-dependent Ca^2+^ or H^+^ transport activity using inside-out PM vesicles as described in Materials and Methods. The ordinate indicates the relative activity of ATP-dependent Ca^2+^ or H^+^ transport. Closed circles; relative activity of ATP-dependent Ca^2+^ transport. Open squares; relative activity of ATP-dependent H^+^ transport. Vertical bars indicate SE.

Figure S2. Estimated PMF before and after light illumination of *Vallisneria* mesophyll cells. (A) Estimated PMF before (Dk) and after illumination with red (RL, 10 µmol m^-2^ s^-1^), blue (BL, 10 µmol m^-2^ s^-1^) and blue (10 µmol m^-2^ s^-1^) plus far-red light (10 µmol m^-2^ s^-1^) (B + FR). PMF was estimated using the measured and published values of membrane potential and cytosolic pH from *Vallisneria* mesophyll cells and gametophyte cells of *Phaeoceros laevis*, listed in Table S1. (B) Effects of 10 µM DCMU on the PMF before (Dk) and after illumination with blue light (BL). 0.5% DMSO, a solvent for DCMU, was used as a control. PMF was estimated using the measured and published values of membrane potential and cytosolic pH from *Vallisneria* mesophyll cells and epidermal cells of *Egeria densa*, listed in Table S1. Vertical bars indicate SE. Each number of data is indicated in Table S1.

Figure S3. Red light-induced hyperpolarization of membrane potential in a *Vallisneria* mesophyll cell*.* A leaf segment was cut open in the middle of the mesophyll layers and a specimen was set in a chamber with the mesophyll cell side upward. The membrane potential (Δ*Ψ*) was measured by glass microelectrode in the specimen kept in complete darkness for 12 to 18 h. The black box in the upper panel indicates a dark treatment. 10 µmol m^-2^ s^-1^ of red light was illuminated (the white box).

Figure S4. Ca^2+^-induced H^+^ efflux from the H^+^-loaded inside-out PM vesicles prepared from *Vallisneria* leaves. After inside-out vesicles had been prepared, ATP-dependent H^+^ uptake into inside-out PM vesicles in the reaction medium containing 2.0 mM ATP was started by addition of 5 mM MgSO_4_. After saturating the reaction, 30 µM CaCl_2_ was added. The addition of CaCl_2_, which is known to inhibit the PM H^+^-ATPase activity [47], induced a rapid collapse of the H^+^ gradient. The subsequent addition of erythrosin B to one of the experiments retarded the rate of decay of the H^+^ gradient. The H^+^ gradient across the membrane of the vesicles was dissipated by nigericin, a H^+^/K^+^ exchanger, to confirm that the membrane vesicles were sealed. The ordinate is the relative intensity of fluorescence from quinacrine.

Figure S5. Inhibitory effects of vanadate with BAPTA or with Ca^2+^ on the ATP-dependent H^+^ transport activity in inside-out PM vesicles prepared from *Vallisneria* leaves. Vanadate with BAPTA and with Ca^2+^ inhibited the activity of ATP-dependent H^+^ transport to 8.5% and 9.2%, respectively. ATP-dependent H^+^ uptake into inside-out vesicles in the reaction medium containing 2.0 mM ATP was started by addition of 5 mM MgSO_4_. After saturating the reaction, the H^+^ gradient across the membrane of the vesicles was dissipated by nigericin, a H^+^/K^+^ exchanger, to confirm that the membrane vesicles were sealed. The ordinate is the relative intensity of fluorescence from quinacrine.
